# High-Throughput Sequencing of Nontuberculous Mycobacterial Flora and *Mycobacterium abscessus* in Cattle

**DOI:** 10.3390/vetsci12030275

**Published:** 2025-03-14

**Authors:** Siqi Chen, Mengda Liu, Yan Li, Jiarui Zhang, Yanfang Li, Yan Liang, Xiaoxu Fan, Yonggang Qu

**Affiliations:** 1 College of Animal Science and Technology, Shihezi University, Shihezi 832003, China; barbatos39@foxmail.com (S.C.); yanfangli@shzu.edu.cn (Y.L.); 18199661608@163.com (Y.L.); 2 Laboratory of Zoonoses, National Animal Brucellosis Specialized Laboratory, China Animal Health and Epidemiology Center, Qingdao 266032, China; liumengda@cahec.cn (M.L.); fanxiaoxu@cahec.cn (X.F.); 3 Xinjiang Production and Construction Corps Animal Husbandry and Veterinary Station, Urumqi 830063, China; bt4641862@163.com (Y.L.); z910181@163.com (J.Z.)

**Keywords:** cattle, nontuberculous mycobacteria, *Mycobacterium abscessus*, epidemiological investigation, high-throughput sequencing analysis

## Abstract

Nontuberculous mycobacteria (NTM) are environmental organisms that can cause opportunistic infections in humans and animals. *Mycobacterium abscessus* (Mab) is a rapidly growing *Mycobacterium* known for its resistance to multiple antibiotics and ability to cause respiratory, skin, and mucosal infections. Understanding the distribution and prevalence of NTM, particularly Mab, in cattle farms and slaughterhouses is crucial for developing effective prevention and control measures. We collected environmental swabs from various surfaces (e.g., feed troughs, sinks, walls, floors, feces, and padding) in cattle farms and slaughterhouses across multiple provinces. High-throughput sequencing technology was utilized to analyze the 16S rDNA V3–V4 region of bacterial DNA extracted from the samples, and qPCR methods were employed to detect and quantify *Mycobacterium abscessus* in the collected samples. Bioinformatics analysis was performed to identify and classify the NTM species present in the samples. This study compared the abundance and diversity of NTM in different environments and assessed the potential zoonotic risk. A total of 1648 environmental swabs were collected from cattle farms and slaughterhouses in 12 provinces of China in 2023, of which 12 samples tested positive for Mab qPCR detection, yielding a detection rate of 0.73% (12/1648). Among them, the detection rate of environmental samples from cattle farms and slaughterhouses was 0.42% (3/720) and 0.87% (9/928), respectively. This study provides valuable information on the epidemiology of NTM in cattle farms and slaughterhouses, contributing to developing effective strategies for preventing and controlling NTM infections. It also enhances our understanding of the zoonotic potential of *Mycobacterium abscessus* and other NTM species.

## Simple Summary

China has a high prevalence of bovine tuberculosis. In addition, nontuberculous mycobacteria infections may impact the quarantine of bovine tuberculosis. This study investigated the contamination status of *Mycobacterium abscessus* in cattle farms and slaughterhouses across 12 provinces in China using high-throughput sequencing technology to examine herds with high positivity rates for caudal-fold skin tests and interferon-gamma release assays. The results indicate that *M. abscessus* contamination was present in the cattle farms and slaughterhouses, and the detection rate of NTM was higher in CFT-positive herds compared to CFT-negative herds, suggesting a potential zoonotic risk. This emphasizes the necessity of enhancing relevant testing protocols for NTM.

## 1. Introduction

Nontuberculous mycobacteria (NTM) are all species of bacteria within the genus *Mycobacterium* (*M.*), except *M. tuberculosis* and *M. leprae*; to date, more than 180 species have been identified [1]. Widely distributed in the environment, NTM predominantly comprise species harmless to humans, but certain strains pose risks as opportunistic pathogens, particularly among immunocompromised individuals [2]. Additionally, some species of mycobacteria are associated with increased mortality in domestic and wild animals or aquatic organisms, contributing to high economic damage and negative environmental impact [3,4]. *M. abscessus* (Mab) is recognized as a significant pathogen capable of causing respiratory, skin, and mucosal infections, with treatment-challenging regimens due to its resistance [2,5].

Since the first isolation of Mab in 1952 and its recognition as a new species of NTM [6], the nomenclature and species/subspecies identification of Mab have undergone multiple revisions. In 1972, following an international collaborative study by the International Working Group on Mycobacterial Taxonomy, Mab was granted subspecies status. In 1992, DNA hybridization technology was used to identify Mab as an independent strain [6,7]. In 2006, based on *rpoB* gene sequence analysis, two new species of Mab subsp. *massiliense* and Mab subsp. *bolletii* were identified. These, along with Mab, form the three subspecies of the *Mycobacterium abscessus* complex (MABC) [8].

MABC poses a great threat due to its high degree of resistance [4,9]. In China, MABC is one of the most common NTM pathogens isolated clinically, accounting for 22.2~23.1% of all NTM strains [10,11,12,13]. Globally, MABC is also the most frequently encountered pathogen in hospitals. Notably, Mab accounts for about 80% of NTM that cause respiratory tract infections. It is more common in immunocompromised populations such as patients with cystic fibrosis (CF), human immunodeficiency virus (HIV)-positive individuals, and those with chronic obstructive pulmonary disease or bronchiectasis. Importantly, Mab has also been detected in the lymph nodes of some wild animals, including a lion that tested positive for *Mycobacterium tuberculosis* [14]. These findings not only suggest Mab as an emerging veterinary pathogen but also highlight its potential interference with the immunodiagnosis of tuberculosis.

Next-Generation Sequencing (NGS) has demonstrated performances comparable to traditional methods in precisely identifying NTM species in clinical specimens, showing its promising potential and efficiency as an alternative tool for the rapid diagnosis of NTM disease [15].

This study investigates the distribution of Mab in Chinese cattle farms and slaughterhouses, aiming to provide a scientific basis for developing effective prevention and control measures. The results are expected to help reduce Mab transmission risks in cattle populations, safeguard livestock industry health, and support public health.

## 2. Materials and Methods

### 2.1. Sample Collection

**Figure 1:**
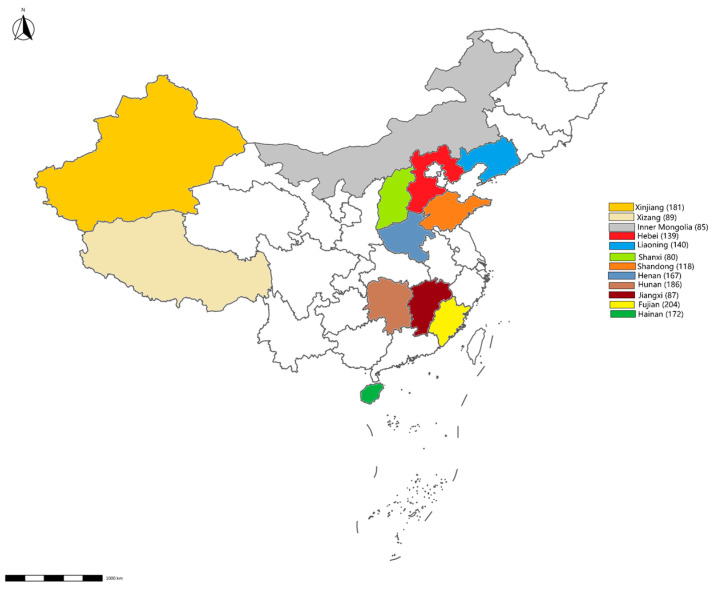
Map showing the sampling sites. The number of samples collected from each province is displayed in brackets.

From January to December 2023, 1648 environmental swabs were collected from 54 cattle farms and 24 slaughterhouses across 12 provinces (Figure 1). These cattle farms and slaughterhouses were selected through stratified random sampling to represent China’s major dairy production regions. The sampling targeted high-contact surfaces (feed troughs, floors, walls) and biological materials (feces, nasal/rectal swabs). After collection, the environmental swabs were placed in 0.5 mL of PSB and stored at −20 °C.

A Xinjiang cattle farm with high caudal-fold skin test (CFT)/interferon-gamma release assay (IGRA) positivity rates was selected for comparative analysis. The CFT and IGRA testing methodology was based on the protocol listed in the Manual of Diagnostic Tests and Vaccines for Terrestrial Animals by WOAH [16]. The cattle were sampled into 4 groups (Table 1). From each group, 20 cattle were randomly chosen, and nasal and rectal swabs were gathered. Specimens were collected using sterile cotton swabs. The dry swab was first moistened with PBS, and the moistened swab was then used to collect cow nasal secretions and rectal feces. The obtained samples were transferred to 5 mL centrifuge tubes containing sterile physiological saline, stored at 4 °C, and transported to the laboratory.

### 2.2. Environmental Samples Detection

The environmental samples were inactivated at 95 °C for 20 min. Then, they were centrifuged, and the qPCR method established by Salvatore et al. [17] was used to detect Mab, with double-distilled water and standard plasmids serving as controls. PCR primers and probes were synthesized by the Beijing Genomics Institute (Beijing, China), and their sequences are detailed in Table 2. All environmental samples were amplified in triplicate on a QuantStudio™ Real-Time PCR System (Thermo Fisher Scientific, Shanghai, China). Real-time PCR amplification was carried out in a 20 μL reaction mixture consisting of 10 μL Premix Ex Taq (Takara, Beijing, China), 15 pM of each primer, and 1 μL of inactivated environmental sample. The cycling conditions were as follows: initial denaturation for 3 min at 95 °C, followed by 15 s at 95 °C, 30 s at 56 °C, and 30 s at 72 °C (40 cycles).

### 2.3. High-Throughput Sequencing of 16S rDNA V3–V4 Region

Total bacterial DNA extraction from nasal swabs and rectal swabs was carried out following the instructions of the QIAamp DNA Stool Mini Kit (Lumiprobe, Shenzhen, China). The concentration of total DNA was determined using NanoDrop2000 (Thermo Fisher Scientific, Shanghai, China) and recorded. The extracted DNA was then sent to Beijing Novogene Company (Beijing, China) for the high-throughput sequencing of the 16S rDNA V3–V4 region. The sequencing primers used were 341F (5′ CCTAYGGGRBGCASCAG-3′) and 806R (5′-GGACTACNNNGGGTATCTAAT-3′). Small fragment libraries were constructed based on the characteristics of the amplified regions, and paired-end sequencing was performed on the libraries using the Illumina NovaSeq (Illumina, San Diego, CA, USA) sequencing platform. The raw sequencing data were demultiplexed based on unique barcodes to separate reads corresponding to each sample, during which the barcodes and primer sequences were trimmed from the reads. Subsequently, the paired-end reads (R1 and R2) were concatenated or merged into longer contiguous sequences using FLASH (1.2.11) software. FastQC (v0.11.9) software was used to perform quality control on the merged tags, obtain clean tags, and filter out chimeric sequences to obtain effective tags for subsequent analysis, and DADA2 was used for denoising and determining each amplicon sequence variant (ASV). Data processing included quality control (FastQC), chimera removal (DADA2), and taxonomic classification (QIIME2, Silva v132).

### 2.4. Specific ASV Screening, Sequence Comparison, and Strain Identification

Based on the amplicon sequence variant (ASV) annotation results, an abundance table was compiled encompassing taxonomic levels from kingdom to species. Subsequently, this study focused on analyzing and screening processes related to the *Mycobacterium* genus from the genus-level abundance table. The representative amplified sequences of these specific ASVs were identified in the corresponding original data, and NCBI-BLAST was utilized for the alignment analysis of these extracted sequences for strain-level identification.

## 3. Results

### 3.1. Detection Results of Environmental Samples

**Figure 2:**
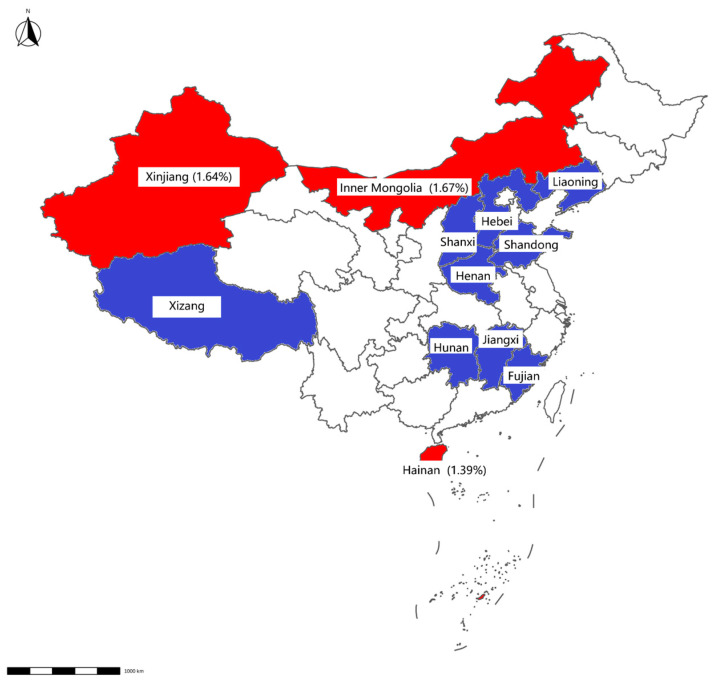
Map displaying detection results of swab samples from cattle farms. The positive rate for each province is displayed in brackets. Provinces with negative Mab results detected by qPCR are highlighted in blue, whereas those with positive results are indicated in red.

**Figure 3:**
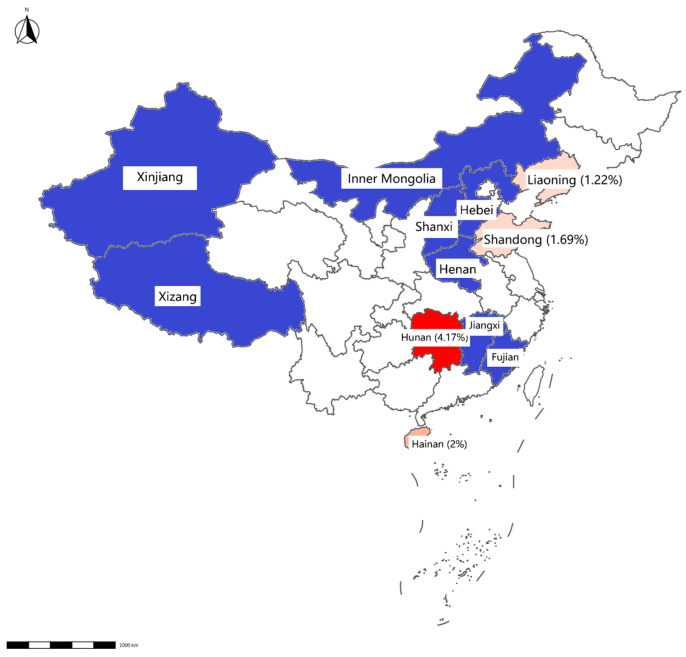
Map displaying detection results of swab samples from slaughterhouses. The positive rate for each province is displayed in brackets. Provinces with negative Mab results detected by qPCR are highlighted in blue, whereas those with positive results are indicated in red.

Mab was detected in 0.73% of samples (12/1648), with higher prevalence in slaughterhouses (0.87%, 9/928) than in farms (0.42%, 3/720). Geographically, Mab-positive samples were clustered in Hainan, Inner Mongolia, Hunan, Shandong, and Liaoning (Table 3, Figure 2 and Figure 3).

### 3.2. Dilution Curve

**Figure 4:**
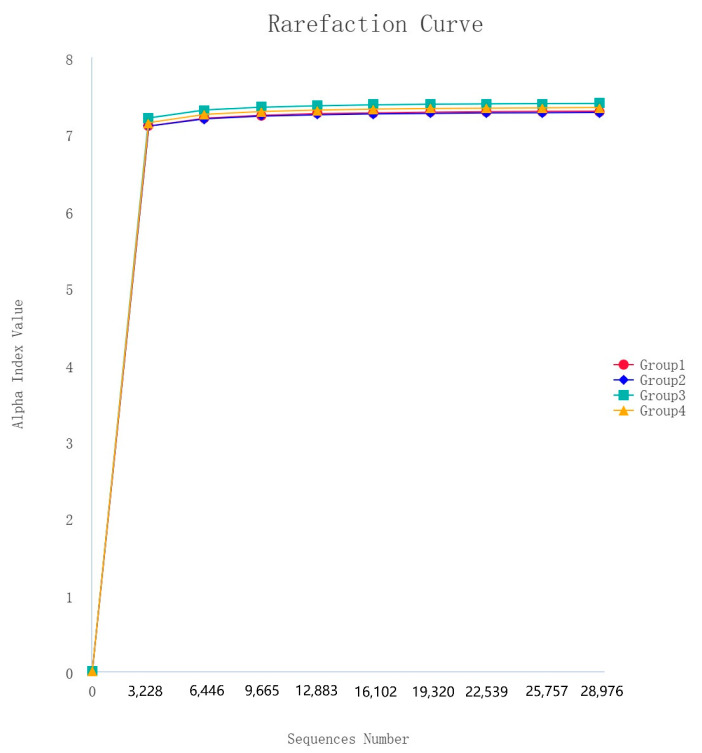
Rarefaction curves of alpha diversity index (Shannon).

After 16S rDNA sequencing, 160 samples underwent quality control, splicing, and noise reduction in the double-ended sequences, resulting in a total of 20,472 ASVs. The observed rarefaction curves for the ASVs and the Shannon alpha diversity index approached those of the saturation phase, indicating good sample coverage, and the asymptotic distribution of the curves suggests that they are comparable (Figure 4).

### 3.3. Analysis of Intergroup Differences in Nasal Swab Samples and Rectal Swab Samples

Beta diversity analysis was carried out on high-throughput sequencing data using QIIME 2.

The linear discriminant analysis effect size (LEfSe) was used to assess the differences between different groups of samples both at the genus or higher taxonomic level.

**Figure 5:**
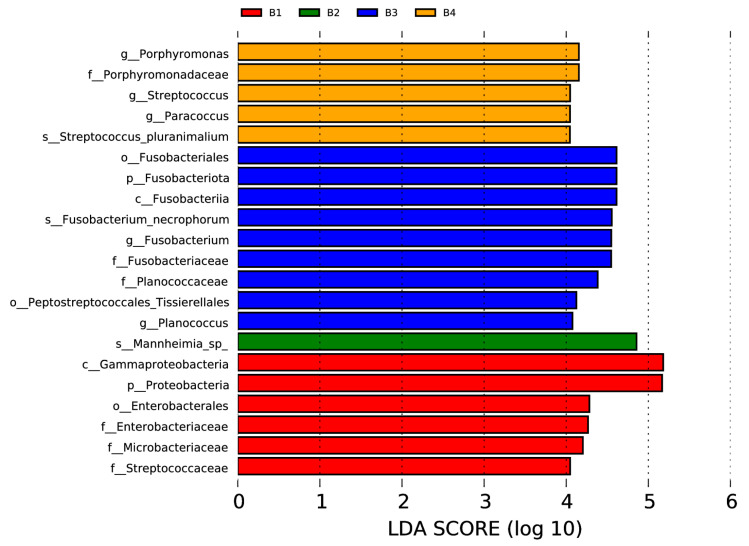
Visualization of most significant taxa (genus or higher level) that differentiate between different groups in bovine nasal swab sample microbiomes.

In the nasal swab samples, there were two phyla (Fusobacteriota and Proteobacteria), two classes (Fusobacteriia and Gammaproteobacteria), three orders (including Fusobacteriales, Peptostreptococcales, Tissierellales, and Enterobacterales), six families (Porphyromonadaceae, Fusobacteriaceae, Planococcaceae, Enterobacteriaceae, Microbacteriaceae, and Streptococcaceae), five genera (*Porphyromonas*, *Streptococcus*, *Paracoccus*, *Fusobacterium*, and *Planococcus*), and three species (*Streptococcus pluranimalium*, *Fusobacterium necrophorum*, and *Mannheimia* sp.) with significant intergroup differences, as shown in Figure 5 (*p* < 0.05, LDA > 4).

**Figure 6:**
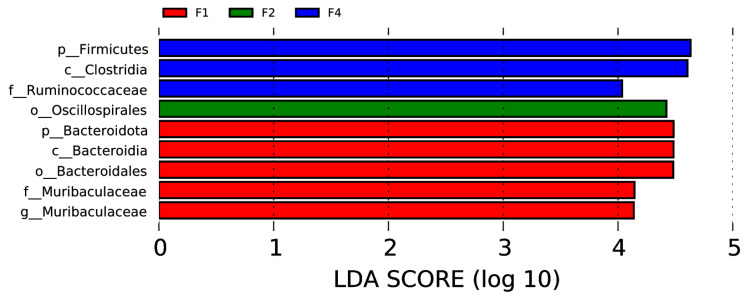
Visualization of most significant taxa (genus or higher level) that differentiate between different groups in bovine rectal swab sample microbiomes.

In the rectal samples, there were two phyla (Firmicutes and Bacteroidota), two classes (Clostridia and Bacteroidia), two orders (Oscillospirales and Bacteroidales), two families (Ruminococcaceae and Muribaculaceae), and one genus (Muribaculaceae) with significant intergroup differences, as shown in Figure 6 (*p* < 0.05, LDA > 4).

### 3.4. Analysis of Mycobacterium ASVs in Different Groups

**Figure 7:**
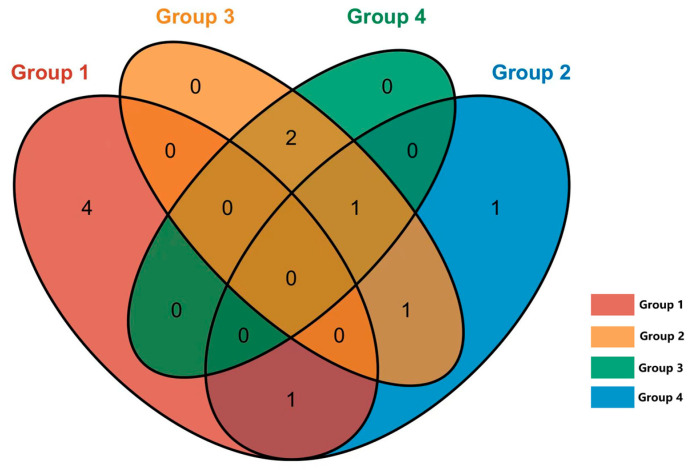
Venn diagram of NTM shared by different groups. Group 1: *M. bovis* positive (IGRA), positive (CFT); Group 2: *M. avium* positive (IGRA), positive (CFT); Group 3: negative (IGRA), positive (CFT); Group 4: negative (IGRA), negative (CFT).

In the nasal and rectal swabs, the genus- and species-level assessments indicated a low abundance of *Mycobacterium* species (less than 0.1% of reads in the sample). These *Mycobacterium* genus-specific reads were isolated manually for further BLAST-based analysis. A total of 40 ASV-representative bacterial genera were identified as *Mycobacterium*. Through NCBI BLAST (https://blast.ncbi.nlm.nih.gov/Blast.cgi, 14 December 2024) comparison, a total of 11 NTM species were identified, with no detection of the *Mycobacterium tuberculosis* complex. The distribution of NTM among different groups is shown in Figure 7.

NTM were detected in 22 out of 60 cows (36.7%) in groups 1–3 (CFT-positive), compared to only 4 out of 20 cows (20%) in group 4 (CFT-negative). Notably, the detection rate of NTM in groups 1, 2, and 3 was significantly higher compared to in group 4.

In group 1, where cows tested positive for CFT and *M. bovis* in IGRA, NTM was detected in 8 cows out of 20, yielding a detection rate of 40%. In group 2, cows that tested positive for CFT and *M. avium* in IGRA, NTM was detected in 7 of the total 20 cows (35%). On the other hand, in groups 3 and 4, where cows tested negative in IGRA, NTM was detected in 12 of the total 40 cows (30%). Notably, there were no statistically significant differences observed in the NTM detection rates among these groups.

In total, 26 nasal swab samples and 3 rectal swab samples tested positive for NTM, revealing that NTM are more likely to be found in the respiratory tract than in the digestive tract. Further analysis through BLAST comparisons identified 11 NTM species: *M. phlei*, *M. aichiense*, *M. abscessus*, *M. saskatchewanense*, *M. smegmatis*, *M. kansasii*, *M. confluentis*, *M. vaccae*, *M. jacquezzii*, *M. celeriflavum*, and *M. stelleraae*.

## 4. Discussion

This study confirms Mab’s presence in Chinese livestock environments, particularly in regions with warm climates and an active animal trade. Xinjiang and Inner Mongolia are two of the main milk supply provinces in China; in these regions the detection rate is lower in slaughterhouses than in livestock farms. However, Hunan and the other provinces mostly supply beef cattle, and in these regions the detection rate in slaughterhouses is significantly higher than that in breeding farms. Our findings reveal significant differences in the NTM contamination on these farms compared to previous reports, which may be attributed to climatic and regional variations [18].

This study sampled 12 major cattle breeding provinces in China. In previous years, multiple studies have shown that the detection rate of *M. bovis* in Xinjiang has always been higher than in other provinces. Therefore, we chose Xinjiang as the main research province and conducted sampling and mNGS analysis on a highly CFT- and IGRA-positive cattle farm in Xinjiang. LEfSe analysis showed distinct microbial communities in the respiratory and digestive tracts across groups. However, these differences mainly resulted from variations in bacterial counts on swabs and were not linked to pathogenic effects. In this study, it appears that cattle infected or latently infected with tuberculosis or NTM do not exhibit a significant effect on the microbiota of their respiratory tract infection. Higher NTM detection rates in CFT-positive herds suggest diagnostic interference, necessitating species-specific testing, but *M. smegmatis* and *M. kansasii* are known to confound tuberculosis tests [19,20]. Although *M. saskatchewanense* and Mab have not been reported as pathogenic to cows [21,22,23], the zoonotic potential of *M. saskatchewanense* and Mab warrants further investigation.

Mab is an important pathogen that causes respiratory, skin, and mucous membrane infections, and its resistance to multiple antibiotics makes it notoriously challenging to treat [2,5]. Due to the lack of widely accessible antibody detection products, this study employed molecular biological techniques to identify the existence of Mab in cattle farms and slaughterhouses. Although at a low detection rate, the presence of Mab was confirmed across numerous provinces in China, and there is a risk of transmission from animals or animal products to humans [24,25]. This report marks the first survey of its kind in the country.

In veterinary medicine, livestock diagnosed with tuberculosis infections are predominantly managed through culling protocols. However, this approach may lead to diagnostic misclassification due to cross-reactivity with infection caused by NTM, imposing substantial economic burdens on global livestock industries. Current evidence suggests that implementing multi-modal diagnostic assays—including interferon-gamma release assays, mycobacterial culture speciation, and molecular differentiation techniques—could effectively distinguish NTM-infected herds from true TB-positive populations [26,27,28].

In veterinary practice, livestock populations misdiagnosed with tuberculosis due to nontuberculous mycobacterial (NTM) infections may be managed through antimicrobial protocols analogous to those in human medicine, typically involving combination therapies with macrolides (e.g., clarithromycin), rifamycins (rifampicin), fluoroquinolones (ciprofloxacin), and aminoglycosides (amikacin) [29,30,31,32,33,34]. Staged culling protocols should be implemented only after confirmed therapeutic failure through mycobacterial culture sensitivity testing and clinical outcome monitoring. This evidence-based approach synergistically preserves productive herds while minimizing zoonotic transmission risks through sustained bacteriological clearance. The transition from blanket depopulation to precision veterinary medicine frameworks enables the maintenance of elite genetic reservoirs and enhancement of herd productivity metrics, concurrently reducing annual agricultural economic losses in affected regions through preserved breeding stock value and optimized antimicrobial stewardship outcomes.

At present, there are many studies indicating that *M. tuberculosis* or *M. bovis* can be transmitted from humans to animals [25,35,36,37,38,39]. In Spain, the United States, and India, the human-to-animal transmission of *M. tuberculosis* or *M. bovis* is well-documented [35,37,40], and most reports on cattle with *M. tuberculosis* are from countries where human *M. tuberculosis* prevalence is very high and likely to have been the source of introduction into the cattle populations [41,42,43]. However, although NTM only poses a potential risk of transmission to humans from animals or animal-derived products [24], there is a lack of research on the transmission of NTM from humans to animals. Unfortunately, the current understanding of NTM’s infectious characteristics, transmission routes, and control strategies in wildlife and livestock remains limited. Given the global rise in zoonotic diseases, Mab represents a significant public health threat. Globalization, climate change, wildlife migration, livestock trade, and human activities may accelerate the spread of NTM. Hence, research on NTM carries not just substantial scientific value but also urgent practical importance.

## 5. Conclusions

Mab and other NTM species pose significant challenges to livestock health and public health. Enhanced surveillance, climate-adapted hygiene protocols, and molecular diagnostics are critical to mitigating transmission risks. This study provides the first national-scale epidemiological data on NTM in Chinese cattle, informing future research and policy development.

## Figures and Tables

**Table 1:** Group information on high-throughput sequencing.

Groups	IGRA Result	CFT Result
Group 1	*M. bovis* positive	Positive
Group 2	*M. avium* positive	Positive
Group 3	Negative	Positive
Group 4	Negative	Negative

**Table 2:** qPCR primer sequences for Mab.

Primer	Primer Sequences (5′-3′)	Primer Length (bp)
Mab 2830-F	CCTCATCGAGGACGGTCAGA	20
2830 abs-Probe	CCGTCGCGAGGCCGGCATCGGCGCACGACGG	31
Mab 2830-R	CACGAATCCGGGCAGCAATA	20

**Table 3:** qPCR detection of Mab in environmental samples from 12 provinces in China.

Province	No. of Samples		Positive Rate of Mab (%)	
	Cattle Farms	Slaughterhouses	Cattle Farms	Slaughterhouses
Fujian	60	144	0.00% (0/60)	0.00% (0/144)
Hainan	72	100	1.39% (1/72)	2.00% (2/100)
Henan	67	100	0.00% (0/67)	0.00% (0/100)
Hunan	66	120	0.00% (0/66)	4.17% (5/120)
Hebei	59	80	0.00% (0/59)	0.00% (0/80)
Shanxi	60	20	0.00% (0/60)	0.00% (0/20)
Shandong	59	59	0.00% (0/59)	1.69% (1/59)
Liaoning	58	82	0.00% (0/58)	1.22% (1/82)
Xinjiang	61	120	1.64% (1/61)	0.00% (0/120)
Inner Mongolia	60	25	1.67% (1/60)	0.00% (0/25)
Xizang	46	43	0.00% (0/46)	0.00% (0/43)
Jiangxi	52	35	0.00% (0/52)	0.00% (0/35)
Total	720	928	0.42% (3/720)	0.87% (9/928)

## Data Availability

The data that support the findings of this study are available from NCBI, reference number PRJNA1120236.
